# APTES-Based Silica Nanoparticles as a Potential Modifier for the Selective Sequestration of CO_2_ Gas Molecules

**DOI:** 10.3390/nano11112893

**Published:** 2021-10-29

**Authors:** Eduardo J. Cueto-Díaz, Alberto Castro-Muñiz, Fabián Suárez-García, Santos Gálvez-Martínez, Mª Carmen Torquemada-Vico, Mª Pilar Valles-González, Eva Mateo-Martí

**Affiliations:** 1 Centro de Astrobiología, (INTA-CSIC), Ctra. Ajalvir, Km. 4, Torrejón de Ardoz, 28850 Madrid, Spain; sgalvez@cab.inta-csic.es (S.G.-M.); mateome@cab.inta-csic.es (E.M.-M.); 2 Instituto de Ciencia y Tecnología del Carbono (INCAR-CSIC), C/ Francisco Pintado Fe, 26, 33011 Oviedo, Spain; alberto@incar.csic.es (A.C.-M.); fabian@incar.csic.es (F.S.-G.); 3 Departamento de Óptica Espacial, Instituto Nacional de Técnica Aeroespacial, Ctra. Ajalvir, Km. 4, Torrejón de Ardoz, 28850 Madrid, Spain; torquemadavm@inta.es; 4 Departamento de Materiales y Estructuras, Instituto Nacional de Técnica Aeroespacial, Ctra. Ajalvir, Km. 4, Torrejón de Ardoz, 28850 Madrid, Spain; vallesgp@inta.es

**Keywords:** functional silica nanoparticles, CO_2_ adsorption, CO_2_/N_2_ selectivity, hybrid nanomaterials, surface spectroscopies

## Abstract

In this work, we have described the characterization of hybrid silica nanoparticles of 50 nm size, showing outstanding size homogeneity, a large surface area, and remarkable CO_2_ sorption/desorption capabilities. A wide battery of techniques was conducted ranging from spectroscopies such as: UV-Vis and IR, to microscopies (SEM, AFM) and CO_2_ sorption/desorption isotherms, thus with the purpose of the full characterization of the material. The bare SiO_2_ (50 nm) nanoparticles modified with 3-aminopropyl (triethoxysilane), APTES@SiO_2_ (50 nm), show a remarkable CO_2_ sequestration enhancement compared to the pristine material (0.57 vs. 0.80 mmol/g respectively at 50 °C). Furthermore, when comparing them to their 200 nm size counterparts (SiO_2_ (200 nm) and APTES@SiO_2_ (200 nm)), there is a marked CO_2_ capture increment as a consequence of their significantly larger micropore volume (0.25 cm^3^/g). Additionally, ideal absorbed solution theory (IAST) was conducted to determine the CO_2_/N_2_ selectivity at 25 and 50 °C of the four materials of study, which turned out to be >70, being in the range of performance of the most efficient microporous materials reported to date, even surpassing those based on silica.

## 1. Introduction

Anthropogenic emission of greenhouse gasses, especially carbon dioxide (CO_2_), has been the center of attention in the media, scientific, and public communities for over four decades [1], reaching an inflection point in 1990, with the Kyoto protocol agreements [2]. Since then, and despite CO_2_ emissions in developed countries having stabilized [3] and COVID-19 pandemic resulting in an extreme drop in daily carbon dioxide emissions, developing countries have doubled it, partially, as a consequence of their growing exports [4]. Indeed, it is estimated that around 80% of China’s power is associated to some extend with fossil fuels [5], while neighboring India generates two thirds of its electricity using coal, and many other Western economies also depend on coal for power generation.

Although many technologies exist, carbon capture storage (CCS) is still one of the most applied technologies for the mitigation of CO_2_ emission. The process involves the separation of CO_2_ from its anthropogenic point sources. In that context, amine scrubbing is one of the most advanced and spread processes, where aqueous solutions of various alkylamines, i.e., monoethanolamine (MEA) [6], remove H_2_S and CO_2_ from the flue gas streams of conventional power plants. Amines react with CO_2_ to form carbamate showing great selectivity over other gases. However they face several limitations as energy requirements are non-negligible [7] and the amine-system is subjected to both corrosion [8] and degradation [9]. Another liquid-based CO_2_ scrubber that is showing great potential are ionic liquids; however, they also display a major drawback as the viscosity increases upon CO_2_ sequestration [10].

This situation has led to extensive empirical search for alternatives where liquid sorbents are replaced by porous-based materials, such as mesoporous silicas [11], metal-organic frameworks [12,13], porous carbon materials [14,15], and zeolites [16,17]. These materials have attracted a considerable interest as they usually possess large surface areas, but also rise several questions regarding their selectivity, adsorption/desorption kinetics, sorbent cost, etc. [18,19,20]. It is evident and undeniable that each material has its own individual limitations hindering their large-scale deployment, i.e., (i) MOFs show outstanding features including easily tunable and tailored structures, well-defined pore properties, and high recyclability [20]; however, they are high-cost materials in terms of production, making them economically unviable, and also their stability is compromised in the presence of moisture [21]. (ii) Porous carbon materials, and in particular, their heteroatom-doped counterparts, have emerged as promising CO_2_ uptake candidates [22,23]. The heteroatom improves the electronegativity of the material enhancing the CO_2_ capture; however, there is an important discrepancy about the CO_2_ binding mechanisms, as it is yet controversial whether the micropore distribution and not the heteroatom doping that determines the CO_2_ capture and CO_2_/N_2_ selectivity [24]. Finally, (iii) SiO_2_ porous materials benefits from their easy surface modification, low energy consumption, considerable adsorption capacity, good selectivity, and tolerance to moisture [25,26,27]; thus, surface modification by amine-based ligands is still a widespread tool for the improvement of the sorption characteristics [28,29,30,31].

**Figure 1:**
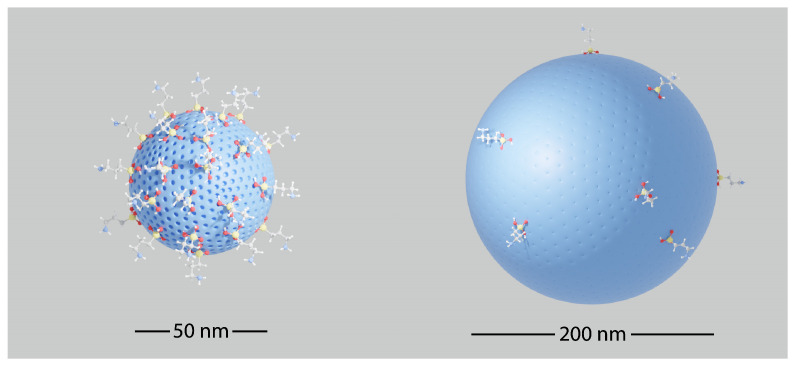
Representation of the two silica-based nanoparticles coated with APTES.

Based on our previous studies in silica nanoparticles (SiO_2_NPs) [32], where a series of amine ligands were covalently anchored on 200 nm size non-mesoporous SiO_2_NPs (SiO_2_ (200 nm)) and their efficiency towards CO_2_ was assessed, we here moved one step further by selecting the most efficient moiety and incorporating it into 50 nm size version of such materials, (SiO_2_ (50 nm)). Thus, in the present work, aminosilane-modified SiO_2_ nanoparticles were synthesized in the presence of (3-aminopropyl) triethoxysilane. The surface properties, morphology, and CO_2_ adsorption/desorption characteristics of the novel 50 nm SiO_2_NPs before and after the modification were studied as well as a comparative between the previously reported 200 nm size and the new nanomaterials (Figure 1).

Finally, and due to their promising characteristics, that will be discussed in this study, i.e., easy functionalization, long-term stability, CO_2_ capture, and outstanding selectivity for CO_2_ gas separation; these hybrid silica nanoparticles could be potential modifiers on NC-CPEs, providing a customized surface for CO_2_ sensing and capture applications.

## 2. Materials and Methods

### 2.1. Materials

For this study, we used: 50 nm size SiO_2_NPs (Nanocym, Scottsdale, AZ, USA). (3-mercaptopropyl) trimethoxysilane (MPTMS) > 96%, 3-aminepropyltriethoxysilane (APTES) > 98%, Ninhydrin 99%, from TCI Europe N. V., Zwijndrecht, Belgium; Toluene 99.8% was purchased from VWR Chemicals, PA, USA and Hexylamine 99% were purchased from Merck Life Science S.L.U., Darmstadt, Germany All chemicals were used without further purification. The gold wafers were acquired from Arrandee metal Gmbh & Co. KG, Werther, Germany.

### 2.2. F3-Aminepropyltriethoxysilaneization of 50 nm SiO_2_ Nanoparticles

**Scheme 1:**
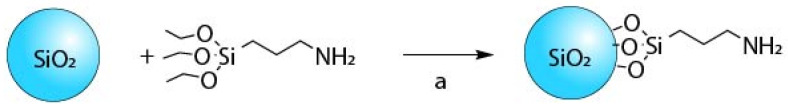
APTES functionalization of SiO_2_ nanoparticles.

The APTES functionalization of the 50 nm size SiO_2_NPs was conducted following and adapting a protocol by Chaix et al. [33], Scheme 1.

A total of 600 mg of SiO_2_NPs were suspended in 25 mL of toluene anhydrous, after that, 1 mL of APTES (4.3 mmol) was added. The reaction mixture was then heated up at 50 °C and kept under stirring for 24 h. After that, the suspension was centrifuged at 10,500× *g* for 10 min, and the supernatant discarded. The crude was exhaustively washed with EtOH and approximately 600 mg of APTES@SiO_2_ (50 nm) was recovered.

### 2.3. Quantification of APTES

**Scheme 2:**
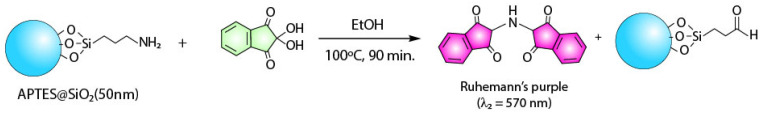
Formation of the Ruhemann’s purple agent.

The quantification of the chemically attached APTES ligands to the silica nanoparticle was assessed by the colorimetric assay, Kaiser test. The overall mechanism involves the monodehydratation of ninhydrin, followed by the formation of the Schiff’s base and the condensation between two ninhydrin-based intermediates through a ketimine bond leading in the Ruhemann’s purple dye (Scheme 2). A calibration curve of the Ruhemann’s dye was recorded using hexylamine solutions in EtOH at different concentrations (showed in the Results Section).

#### Kaiser Test on APTES@SiO_2_ (50 nm)

Following the procedure of Poli et al. [34], 6.6 mg of APTES@SiO_2_ (50 nm) and 1 mL of the ninhydrin solution were introduced in a sealed reaction glass tube, and diluted in EtOH up to a total volume of 6 mL. The mixture was heated up at 100 °C and kept under stirring for 90 min, after that the suspension was cooled down, centrifuged at 10,500× *g* for 15 min, and the supernatant was analyzed by UV-Vis.

### 2.4. Surface Characterization

#### 2.4.1. XPS

X-ray photoelectron spectroscopy (XPS) analysis of the samples was carried out in an ultra-high vacuum chamber equipped with a hemispherical electron analyzer and with the use of an Al Kα X-ray source (1486.6 eV) with an aperture of 7 × 20 mm. The base pressure in the chamber was 5 × 10^−10^ mbar, and the experiments were performed at room temperature. The peak decomposition in different components was shaped, after background subtraction, as a convolution of Lorentzian and Gaussian curves. Binding energies were calibrated against the binding energy of the Au 4f_7/2_ peak at 84.0 eV for the gold samples. With the aim of analyzing the SiO_2_ (50 nm) and APTES@SiO_2_ (50 nm) elemental composition and chemical bonding, the nanoparticles were immobilized in a 11 × 11 × 1 mm size gold wafer following a protocol adapted from Cueto et al. [35]. To a solution of 3-MPTS (40 mM) in MeOH was added a gold wafer and was kept submerged for 3 h. After that, the wafer was rinsed with MeOH, dried, and submerged in a different solution of NaOH 0.01 M for 3 h. In parallel, a pH = 9 suspension of SiO_2_ (100 mg) in 20 mL of mQ water was prepared. The suspension was sonicated for 10–15 min, and a few drops were placed over the Au wafer bearing the linker until dryness. The unreacted SiO_2_NPs were removed by consecutive rinsing with EtOH and H_2_O.

#### 2.4.2. Infrared Spectroscopy

The samples were recorded in a Nicolet IS50 (ThermoFisher Scientific, USA) equipped with a DLaTGS detector and a XT-KBr beamsplitter. Sample aliquots of approximately 10 mg of both SiO_2_ (50 nm) and APTES@SiO_2_ (50 nm) were placed onto the ATR diamond and each spectrum was recorded without additional sample preparation and by accumulating 64 scans in the 4000–500 cm^−1^ spectral range with a resolution of 0.1 cm^−1^.

#### 2.4.3. SEM Microscopy

The hybrid nanomaterials were immobilized into Au wafers following the protocol adapted from Cueto et al. [35] and recorded in a ThermoScientific Apreo C-LV field emission electron microscope (FE-SEM) equipped with an Aztec Oxford energy dispersive X-ray microanalysis system (EDX)

#### 2.4.4. AFM Microscopy

Surface morphology of the materials was measured by atomic force microscopy (AFM) using a XE-150 SPM/AFM (Park Systems Corp., Suwon, Korea), operating in air at room temperature. True Non-Contact ModeTM provided high-resolution images of SiO_2_ nanoparticles and allowed surface preservation. Heavily doped silicon tips (910M-ACTA) with aluminum coating 30 nm thick (force constant 40 N/m, resonance frequency 300 kHz) were used. The tip radius of curvature reported by the manufacturer is less than 10 nm. Images were recorded at a scan rate of 0.3 Hz and a resolution of 256 × 256 pixels. For a 3 × 3 micron^2^ scan area, one pixel in a 256 × 256 image corresponds to an area of 11.7 × 11.7 nm. XEI program was used for image processing and measurements of the acquired data. Samples were analyzed without any pre-treatment.

### 2.5. Thermogravimetric Analisys (TGA)

TGA were carried out in a MK-M5 microbalance (CI Electronic, Salisbury, UK) under an air flow of 50 cm^3^/min with a heating program of 10 °C/min from room temperature up to 950 °C.

### 2.6. CO_2_ Adsorption and N_2_ Adsorption Isotherms

CO_2_ adsorption isotherms of the samples at 0, 25, and 50 °C and N_2_ adsorption isotherms at 25 and 50 °C were measured in a volumetric device Autosorb-1 (Quantachrome) in the 10^−5^ bar to 1 bar pressure range (gas purity; N_2_: 99.9996%, CO_2_: 99.995%). From the CO_2_ adsorption isotherms at 0 °C, the narrow micropore volume (pore width smaller than about 0.7 nm) was obtained by applying the Dubinin-Radushkevich equation [36] in the 10^−4^ to 0.03 P/P_0_ range. The isosteric heat of adsorption was calculated by applying the Clausius-Clapeyron equation to the adsorption isotherms measured at the afore mentioned three temperatures and also applying the Virial equation, simultaneously, to two couples of isotherms (isotherms at 0 and 25 °C, and isotherms at 25 and 50 °C) following the methodology proposed in the bibliography [37,38]. More details are included in ESI (Figure S1). Samples (c.a. 250 mg) were degassed at 150 °C for 18 h under vacuum conditions before each sorption measurement, with the aim of eliminating the sample humidity and any other adsorbed gases. The porous texture of the materials was probed by physical adsorption/desorption of N_2_ at −196 °C in a volumetric device (ASAP 2010 from Micromeritics). The samples were degassed under the same conditions as described above. The surface area, S_BET_, was calculated by the Brunnauer-Emmet-Teller (BET) equation from the N_2_ adsorption data in the relative pressure range of ca. 0.015–0.25, the external surface area, S_ext_, was obtained by applying the t-plot method to the adsorption branch and the total pore volume V_T_, was obtained by the Gurvich rule from the amount of adsorbed N_2_ at the relative pressure of 0.95. The micropore volume, V_DR_, _N2_, was calculated by applying the Dubinin-Radushkevich method at P/P_0_ < 0.1, and the mesopore volume, V_mp_, was estimated as V_mp_ = V_T_ − V_DR_, _N2_ [39].

### 2.7. Adsorption Selectivity of Binary Mixtures of CO_2_/N_2_

The adsorption selectivity of CO_2_ over N_2_ on the SiO_2_ nanoparticles both pristine and functionalized were estimated by applying the Ideal Adsorbed Solution Theory (IAST) proposed by Myers and Prausnitz [40] to the pure CO_2_ and N_2_ adsorption isotherms measured at 25 and 50 °C. The detailed thermodynamic deduction of the IAST theory can be found in the original work of Myers and Prausnitz [40] and many other articles [41,42]. Here, we briefly summarized IAST to introduce the main equation and define the notation. IAST allows to predict the amount of each gas adsorbed on the surface of an adsorbent in equilibrium with a multicomponent gaseous mixture. The following assumptions are made for the isothermal adsorption process: (1) the adsorbent is thermodynamically inert (i.e., the change on its thermodynamic properties is negligible compared to the change in the same property of the adsorbate); (2) the surface area of the adsorbed is invariant for all gases (i.e., the adsorbed gases have access to the same area of the adsorbent); and (3) the Gibbs definition of adsorption applies for the process.

With the assumptions of this adsorption model, the thermodynamic equations can be written for the adsorbed phase by changing the volume for area *A*, and the pressure for the spreading pressure *π*, which is the analog to pressure in two dimensions. Thus, for example, the Gibbs free energy, *G*, of the adsorbed phase is defined as:

(1) $$dG=-SdT+Ad\pi +\sum_{i=1}^{N}\mu_{i}dn_{i}$$

where, *n_i_* is the moles of gas adsorbed in the surface area, *A*, at the spreading pressure, *π* and *µ_i_* are the chemical potential of the adsorbed phase.

The spreading pressure cannot be measured, but can be calculated from adsorption isotherms of the pure gases (Equation (2)):

(2) $$\pi_{i}^{0}=\frac{RT}{A}\int_{0}^{P_{i}^{0}}\frac{n_{i}}{P_{i}}dP_{i}$$

where, $P_{i}^{0}$ is the partial pressure of pure component *i* at the spreading pressure and temperature of the mixture. The integral fitting in Equation (2) can be calculated by two different methods: (i) by fitting the experimental adsorption isotherms data to a model from which an analytical solution can be found or (ii) by a numerical calculation. Here, we used the second method. In the case of an ideal solution, the partial pressure is calculated using an analog to Raoult’s law (Equation (3)):

(3) $$P_{i}=y_{i}P=x_{i}P_{i}^{0}\left(\pi \right)$$

*y_i_* and *x_i_* are the mole fraction of component *i* in the gas and adsorbed phases, respectively.

Additionally, the following relations are followed for an ideal solution where the mixing process is carried out at constant spreading pressure, *π*:

(4) $$\sum_{i=1}^{N}x_{i}=1$$

(5) $$\sum_{i=1}^{N}y_{i}=1$$

(6) $$\pi =\pi_{1}^{0}=\pi_{2}^{0}=\cdots =\pi_{N}^{0}$$

In the case of a binary mixture, there are nine unknowns and seven independent equations. Thus, to calculate all the variables, two of them have to be set (for example, *P* and *y*_1_). The procedure to solve IAST consists of the following steps: (i) to calculate the spreading pressures for each component from the pure isotherms, (ii) at a given *P* and *y*_1_, $P_{i}^{0}$ at the same spreading pressures (Equation (6)) for each component are determined, and (iii) the other variables are calculated by using Equations (3)–(5).

The total adsorbed amount, *n_T_*, is calculated from the pure isotherms taking into account the mole fractions of each component in the adsorbed phase, *x_i_* (Equation (7)):

(7) $$\frac{1}{n_{T}}=\sum_{i=1}^{N}\frac{x_{i}}{n_{i}^{0}}$$

The adsorption isotherm of each component in the mixture can be calculated by Equation (8):

(8) $$n_{i}=x_{i}n_{T}$$

Finally, the selectivity coefficient of component *i* over *j* is defined by the following Equation (9):

(9) Si,j=xiyixjyj=Pj0Pi0

## 3. Results

Typically, at nanoscale, particles exhibit many thermo-physical features distinct from those found at the microscale. Therefore, as the size decreases, the increased surface-to-volume ratio and the associated higher surface energy enhance the reactivity of most nanoparticles. In our case, a comparison study between 50 and 200 nm was needed.

### 3.1. Characterization of the Nanomaterials

#### 3.1.1. Colorimetric Assay

**Figure 2:**
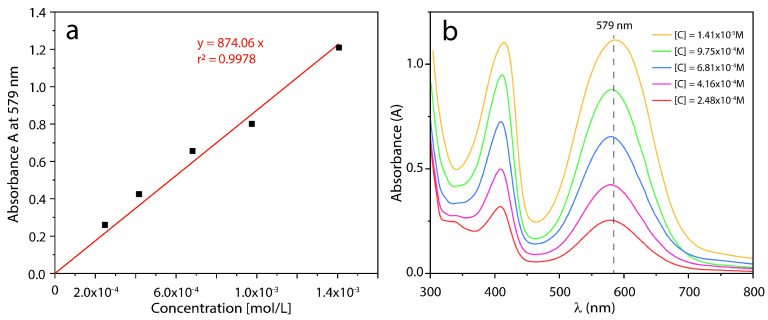
(**a**) Calibration curve; (**b**) absorption spectra of the different hexylamine solutions conducted by the Kaiser test.

The preparation of the hexylamine calibration curve (Figure 2a) was performed as follows: 

A stock solution of hexylamine and ninhydrin were prepared at a concentration of 1.43 × 10^−2^ M and 1.56 × 10^−2^ M, respectively, keeping the ninhydrin solution in the dark during the course of the analysis. Analyte solutions containing hexylamine concentrations ranging from 2.48 × 10^−5^ M to 1.41 × 10^−3^ M were freshly prepared (0.1 to 0.5 mL of the hexylamine solution, 1 mL of the ninhydrin solution, and absolute ethanol up to a total volume of 6 mL were introduced in each sample). The flasks were sealed, stirred for 90 min at 100 °C, and the UV-Vis spectroscopy experiments (Figure 2b) were conducted once the solutions were cooled down.

**Figure 3:**
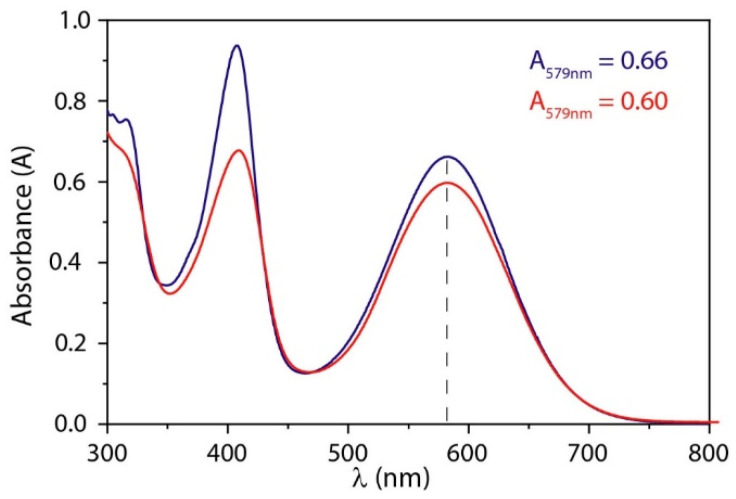
Absorption spectra of APTES@SiO_2_ (50 nm) and APTES@SiO_2_ (200 nm) treated with ninhydrin.

Kaiser tests were then conducted in the two samples bearing the unknown concentration of APTES, (i) APTES@SiO_2_ (200 nm), and (ii) APTES@SiO_2_ (50 nm) (Figure 2 and Scheme 2). The tests were performed as follows: (i) 60.2 mg of APTES@SiO_2_ (200 nm) was suspended in 6 mL solution EtOH/ninhydrin (5:1) stirred for 90 min at 100 °C and the UV-Vis spectrum was recorded of the filtered solution (Figure 3, red); similarly (ii) 6.6 mg of the APTES@SiO_2_ (50 nm) was suspended in 6 mL solution EtOH/ninhydrin (5:1) stirred for 90 min at 100 °C and the UV-Vis spectrum was recorded of the filtered solution (Figure 3, navy blue).

The recorded Absorbance at λ = 579 nm for the Ruhemann’s purple were 0.60 and 0.66 respectively, which corresponds to concentrations of (C)_APTES_ = 6.85 × 10^−4^ M for APTES@SiO_2_ (200 nm) and (C)_APTES_ = 8.55 × 10^−4^ M for APTES@SiO_2_ (50 nm). Resulting in coverages of 69 μmol (APTES)/g SiO_2_ and 780 μmol (APTES)/g SiO_2_ respectively, we observe a clear improvement in the grafting capacity of the nanoparticles, which was expected as the effective surface per nanoparticle is four times larger and the surface energy is increased.

#### 3.1.2. X-ray Photoelectron Spectroscopy

**Figure 4:**
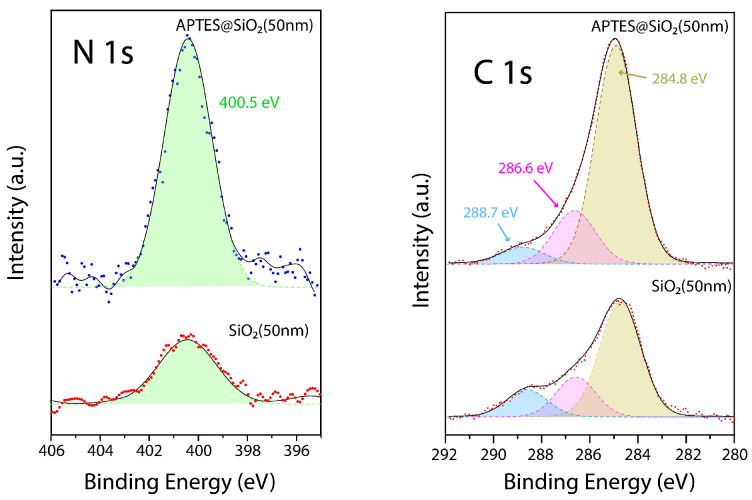
XPS spectra of the (**left**) N 1s region and (**right**) C 1s for the SiO_2_ (50 nm) and APTES@SiO_2_ (50 nm) samples.

A XPS analysis of the SiO_2_ (50 nm) nanoparticles before and after APTES adsorption was performed in order to confirm the success of the APTES grafting on the silica NPs. As presented in Figure 4, C 1s and N 1s core levels peaks were compared before (SiO_2_ (50 nm)) and after (APTES@SiO_2_ (50 nm)) APTES functionalization.

XPS spectra of the N (1s) region (Figure 4, left) shows a remarkable increase of the nitrogen intensity after APTES functionalization process, deconvolution of the signal shows only one component at a binding energy of 400.5 eV, which is attributed to NH_2_ groups [43,44], due to the chemical composition of the APTES molecule. A deconvolution study of the C 1s peak (Figure 4, right) shows three components for both cases, the first component has a binding energy (B.E.) at 284.8 eV is attributed to the C–H and C–C group [35], the second component at 286.6 eV corresponds to O–CH_3_ (MPTS-mediated binding by Au wafers to NPs) and C–N groups (APTES chemical composition), whereas the third component observed at 288.7 eV is assigned to the C=O groups. After APTES functionalization there is a remarkable increase, four times the intensity for the first component (at 284.8 eV, assigned to the C–H and C–C group [35]), and three times the intensity for the second component at 286.6 eV related to the C–N groups, both carbon components are strongly related to the functional groups forming part of APTES ligand structure. Then, the increment of the XPS signal for both elements, nitrogen and carbon, confirm the successful linkage of the APTES molecule onto the silica nanoparticles’ surface.

#### 3.1.3. ATR-FTIR

**Figure 5:**
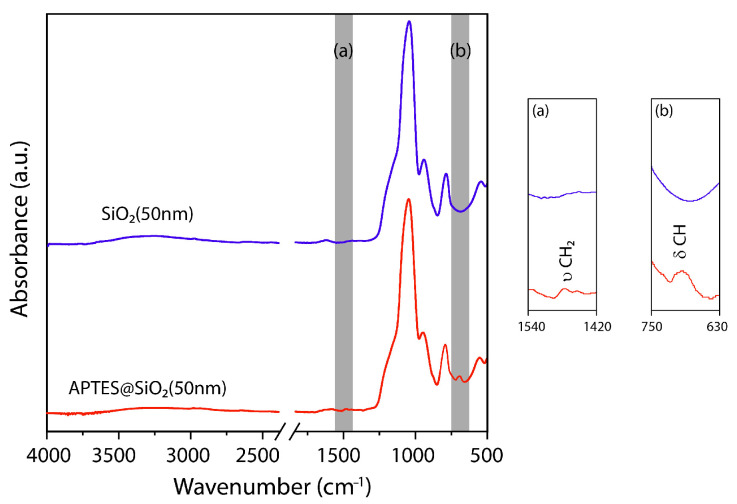
IR spectra of 50 nm size SiO_2_ nanoparticles, before (blue) and after (red) APTES functionalization. (insets) zoomed-in FTIR spectra of the vibrational modes of –NH_2_ and –CH_2_ (**a**), and –CH (**b**).

IR spectra (Figure 5) of both SiO_2_ (50 nm) and APTES@SiO_2_ (50 nm) samples are governed by a broad band (950–1250 cm^−1^) with a maximum at 1050 cm^−1^, which corresponds to the Si–O–Si bonds [45]. However, after APTES functionalization, two new bands appears at 1490 (Figure 5a) and 690 cm^−1^ (Figure 5b), being ascribed to bending vibrations of –NH_2_ [46] and –CH_2_ and deformation of –CH out of plane [47]. These results are in agreement with a successful SiO_2_ modification after APTES functionalization.

#### 3.1.4. TGA Analysis

**Figure 6:**
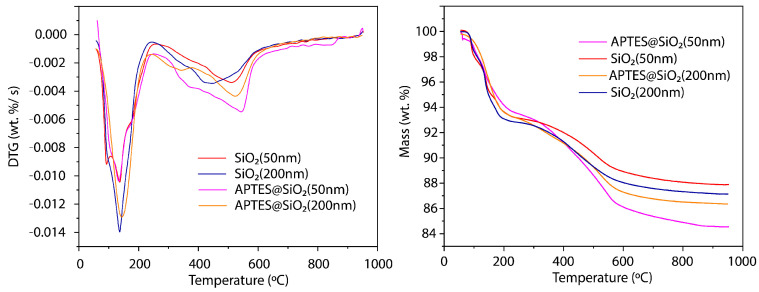
Thermal analysis (**left**) DTG and (**right**) TGA for SiO_2_ (50/200 nm) and hybrids SiO_2_ (50/200 nm)@APTES.

The relative thermal stability of the nanoparticles was evaluated by thermogravimmetry. The TGA-DGT and their derivative (DGT) curves (Figure 6) were conducted from room temperature to 950 °C and are depicted in Figure 5. It is interesting to notice quite similar degradation patterns in the four cases: SiO_2_ (50 nm), SiO_2_ (200 nm), APTES@SiO_2_ (50 nm), and APTES@SiO_2_ (200 nm). A mass loss in the temperature range of 50–180 °C is approximately 7 wt %, attributed to the evaporation of physically adsorbed water. Then, a weight loss from 200–600 °C is mainly due to the condensation of silanol groups to give siloxane through the loss of a water molecule [48,49]. In the case of functionalized samples, the decomposition of the aliphatic chains occurs in the same interval of temperatures. The weight loss is higher in the sample APTES@SiO_2_ (50 nm) than in its counterpart APTES@SiO_2_ (200 nm), which is in agreement with the greater amount of APTES that was grafted in the former one, and previously demonstrated by colorimetry. Above 600 °C and up to 950 °C, a light and very constant weight loss is observed and attributed to residual condensation of silanol groups.

Regarding the weight loss of functionalized vs. un-functionalized pristine silica nanoparticles, it is clear the increase of weight loss in the 50 nm series, APTES@SiO_2_ (50 nm) vs. SiO_2_ (50 nm), compared to their 200 nm counterparts. This is also in agreement with the coverage values obtained with the colorimetric analysis.

#### 3.1.5. Morphology of the Nanoparticles

**Figure 7:**
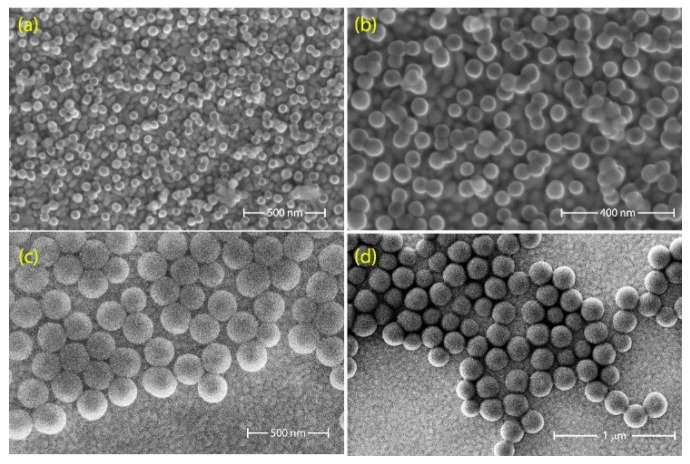
SEM micrographs of the 50 nm size nanoparticles (**a**,**b**) and 200 nm size SiO_2_ immobilized over Au wafers (**c**,**d**).

**Figure 8:**
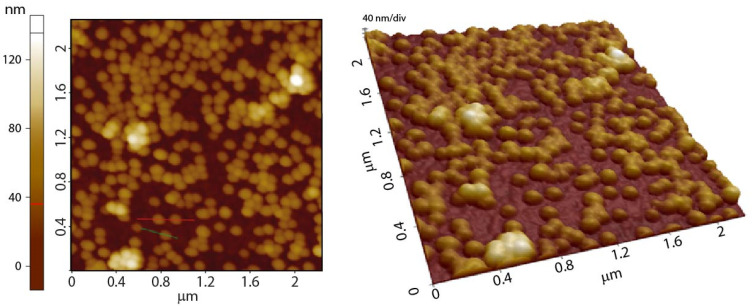
AFM topography micrographs of SiO_2_ (50 nm) deposited over Au wafers.

The morphology of the nanoparticles as well as the immobilization details were investigated by both Scanning Electron Microscopy (SEM, Figure 7) and Atomic Force Microscopy (AFM, Figure 8).

As shown in Figure 7, the SEM micrographs displayed the silica nanoparticles as nanoobjects of 50 nm size, exhibiting a typical nanosphere morphology with smooth surface and homogeneous diameter. 

In addition to SEM, Atomic Force Microscopy (Figure 8) was conducted in the same sample. Here, the average size measured by AFM was similar to SEM (40–50 nm); also, the smoothness and dispersity of the nanomaterial was assessed displaying similar results as for the SEM studies. Nevertheless, due to the axial (z)-displacement of the piezoelectric, the height of SiO_2_ (50 nm) was measured and found to be relatively constant at 40–50 nm.

#### 3.1.6. Textural Characterization of the SiO_2_ (50 nm) and SiO_2_ (200 nm)-Based Nanoparticles

**Figure 9:**
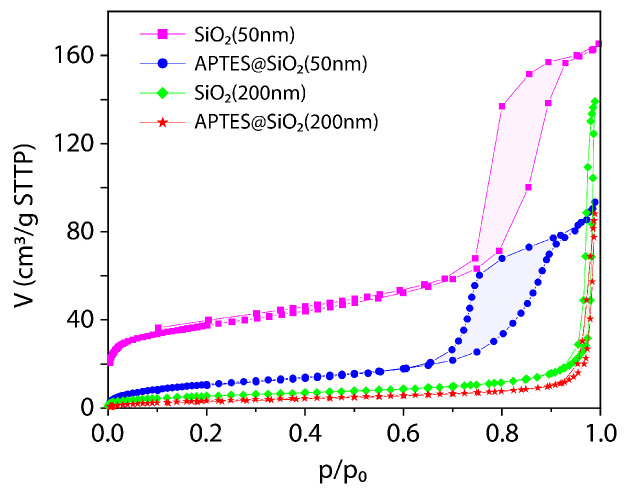
N_2_ adsorption isotherms at −196 °C for the four cases subject of study.

The N_2_ isotherms for both the SiO_2_ (50 nm) and APTES@SiO_2_ (50 nm), and for comparative issues SiO_2_ (200 nm) and APTES@SiO_2_ (200 nm), are displayed in Figure 9. The two first ones (50 nm) showed typical type IV isotherms with H2 hysteresis loop, which is characteristic of mesoporous adsorbents, whereas the 200 nm size nanoparticles displayed a type II isotherm, distinctive of non-porous materials. It is expected that these materials do not have pores and that the adsorption takes place on the outside of the nanoparticles. Nevertheless, the theoretical specific surface areas of silica nanospheres are 200 and 50 nm in diameter, assuming a density of 2.65 cm^3^/g are 11 and 45 m^2^/g, respectively. These values are about 40% lower than the external surface areas obtained by applying the t-plot method to the adsorption data (see Table 1), indicating that the surface of the silica nanoparticles might show certain roughness or porosity. The decrease in the nanoparticle size produces an expected increase in area (in this case the BET surface area increases from 20 to 129 m^2^/g and the external surface area S_ext_, from 20 to 76 m^2^/g, as seen in Table 1) due to the increase in the area/volume ratio of the nanoparticles as their diameter decreases. The increase in the amount adsorbed at pressures >0.6 for particles of 50 nm in size and from 0.95 for those of 200 nm, is produced by the condensation of nitrogen in the space that remains between the aggregates of the nanoparticles. As the particle size decreases, the distance between them decreases, causing condensation to occur at lower pressures and the appearance of hysteresis cycles due to the separation being the size of the mesopores.

Grafting with APTES means that the N_2_ adsorption capacities decrease due to the ligands having occupied part of surface of the silica nanoparticles. This decrease is more pronounced for the 50 nm than for its 200 nm counterpart. Thus, when functionalizing the nanoparticles, the area decreases from 129 to 41 m^2^/g for those of 50 nm and from 20 to 13 m^2^/g for those of 200 nm. These S_BET_ for the functionalized nanoparticles are very similar to the S_ext_ ones (Table 1) and very close to the theoretical values (11 and 45 m^2^/g for nanoparticles of 200 and 50 nm in diameter, respectively), suggesting that the functionalization smooths the surface of the silica nanoparticles and blocks any possible porosity that may exist. This effect is more pronounced for the 50 nm nanoparticles because of the more efficient APTES functionalization (780 μmol (APTES)/g SiO_2_) compared to the 200 nm (69 μmol (APTES)/g SiO_2_). The total pore volume (V_T_) and the micropore volume (V_DR_, _N2_) displayed a similar trend in both assessments. V_DR_, _N2_ was also reduced after functionalization of the silica nanoparticles, although the decrease is lower due to the higher affinity of the APTES-amine groups for the CO_2_ gas molecules.

### 3.2. CO_2_ Adsorption Studies

**Figure 10:**
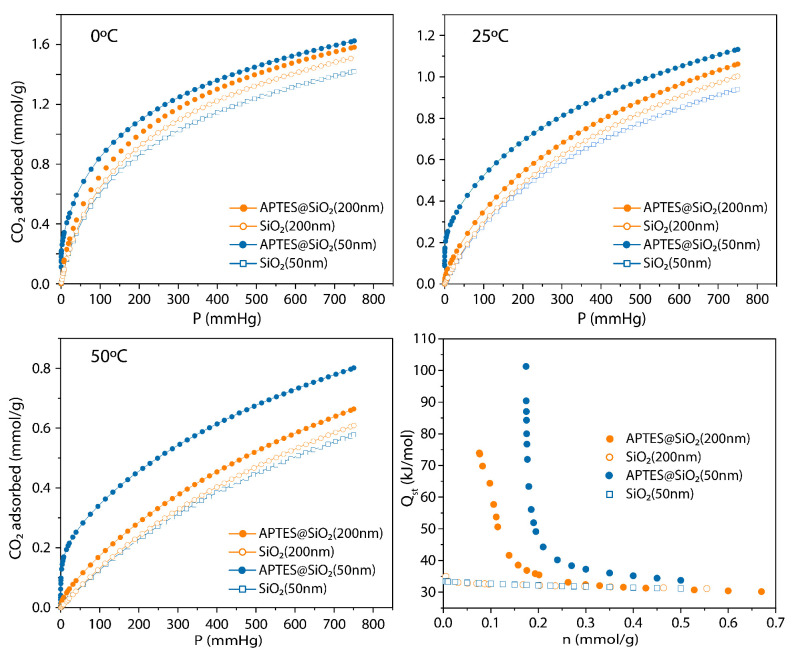
CO_2_ adsorption isotherms at 0, 25, and 50 °C for pristine and functionalized SiO_2_ nanoparticles of 50 and 200 nm. Isosteric heat of adsorption (Q_st_).

**Figure 11:**
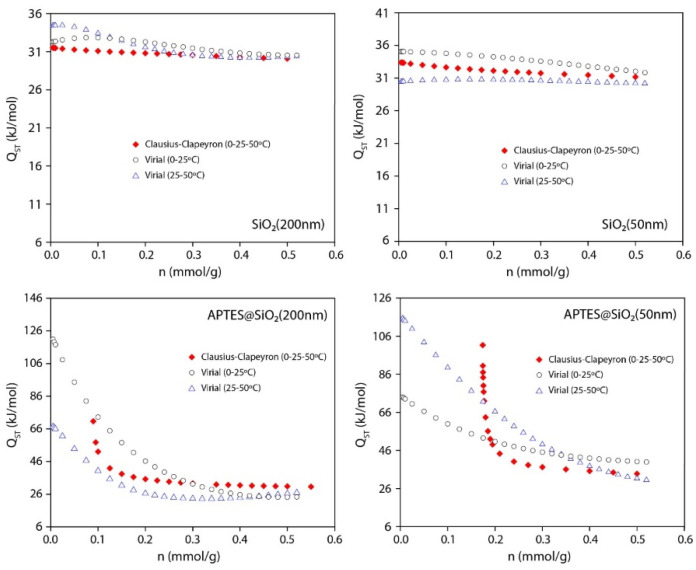
Heat of adsorption from Clausius-Clapeyron (red) using the isotherms at the three temperatures and from Virial equation fitting simultaneously the isotherms at 0 and 25 °C (black) and at 25 and 50 °C (blue).

The four cases of study were tested as sorbents for CO_2_ capture at three different temperatures, 0, 25 and 50 °C (Figure 10). As expected, when increasing the temperature, the CO_2_ uptake capacities dropped in each of the samples; however, this decrease is much less marked in the particular case of APTES@SiO_2_ (50 nm). This phenomenon can be ascribed to the existence of strong interactions between the CO_2_ gas molecules and the APTES-amine groups, showing heats of adsorption of the order of those expected in chemisorption processes, as can be seen in Figure 10. The two functionalized samples have very high heats of adsorption calculated by the Clausius-Clapeyron equation at low coverage degrees >75 kJ/mol for APTES@SiO_2_ (200 nm) and >100 kJ/mol in the case of APTES@SiO_2_ (50 nm), which strongly decrease with the amount of CO_2_ adsorbed, resulting in similar values (~31 kJ/mol) to those of the non-functionalized samples. Similar results were obtained by Virial equations (Figure 11). Pristine SiO_2_ nanoparticles presented similar heat of adsorption regardless of the method and the isotherms used for the calculation. In the case of the functionalized samples, the heat of adsorption obtained with the Virial equation, is directly linked to the couple of isotherms selected for the study. This can be ascribed to the high dependance that this method requires for the achieved fitting. In any case, even when taking the lowest values obtained by this method, the heat of adsorption at low coverage degrees of about 67 kJ/mol for APTES@SiO_2_ (200 nm) and 74 kJ/mol for APTES@SiO_2_ (50 nm). Despite these high values, the desorption is completely reversible and no hysteresis was observed in any of the samples (Figure S2), except for a very small one in the particular case of APTES@SiO_2_ (50 nm), which can be attributed to the higher amount of APTES-amine groups present in the nanoparticle’s surface, thus leading to strong interactions between the CO_2_ gas molecules and the grafted APTES, promoting a delay in the desorption process. On the other hand, these very high heats of adsorption are responsible for the high selectivity towards CO_2_ adsorption, as will be shown later in the CO_2_/N_2_ separation studies and are also responsible for the fact that these samples keep a high CO_2_ adsorption at high temperatures.

**Figure 12:**
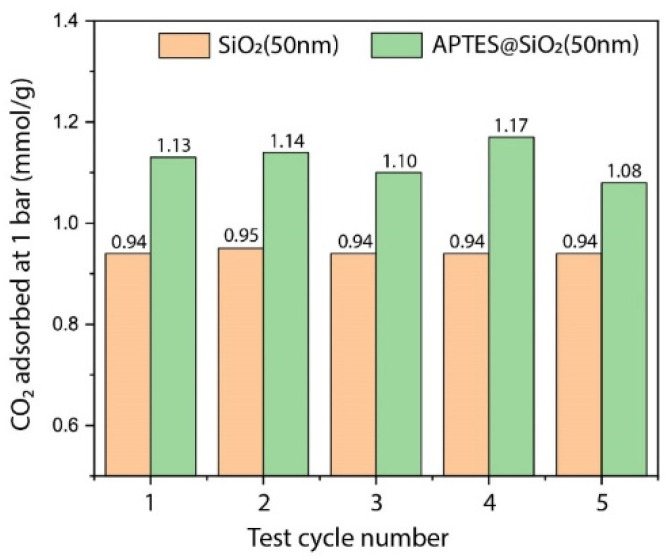
Stability of pristine and functionalized 50 nm SiO_2_ nanoparticles under repeated adsorption-desorption cycles at 25 °C and up to 1 bar.

However, despite presenting such a very high heat of adsorption values, their regeneration could be carried out simply by means of vacuum at room temperature. As can be seen in Figure 12, the samples maintain their adsorption capacity in five consecutive adsorption/desorption cycles. Each cycle consisted of an adsorption/desorption isotherm and between each one of them no degassing step was carried out, simply, the vacuum stage that is programmed in the procedure for measuring adsorption isotherms of the device (a few minutes at room temperature) was used. To assess the stability of the 50 nm size samples SiO_2_ (50 nm) and APTES@SiO_2_ (50 nm) after five cycles, FT-IR spectra was conducted right after the experiments. Figure S3 showed no apparent chemical modification in the surface of the nanosphere; thus, confirming the good stability of the samples.

**Figure 13:**
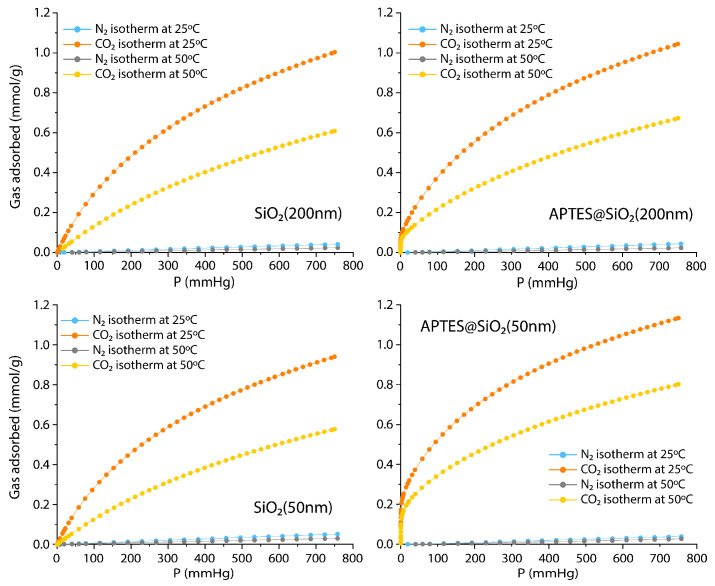
CO_2_ and N_2_ adsorption isotherms at 25 and 50 °C on pristine and functionalized SiO_2_ nanoparticles of 50 and 200 nm size.

The adsorption selectivity of CO_2_ over N_2_ was determined by applying the IAST to the pure adsorption isotherms of both gases at two temperatures (25 and 50 °C). The fitting of the experimental adsorption isotherm data to an analytical model gave rise to significant errors in the case of the modified silica nanoparticles, what might lead to incorrect predictions. Therefore, the spreading pressures were calculated from Equation (2) by numerical integration [42]. Figure 13 shows the CO_2_ and N_2_ adsorption isotherms at 25 and 50 °C on the four samples. Similar to the case of CO_2_, the amount of adsorbed N_2_ decreased when increasing the adsorption temperature, as it is expected in a physisorption process. All samples have a much lower N_2_ adsorption capacities than CO_2_ adsorption. Furthermore, the N_2_ adsorption isotherms have a lineal shape, where the adsorbed amount increases linearly with the pressure, following Henry’s law. The low value of Henry’s constants (see Table S1 in Supporting Information) proves the poor interaction between N_2_ and the adsorbents given almost negligible adsorption. 

**Figure 14:**
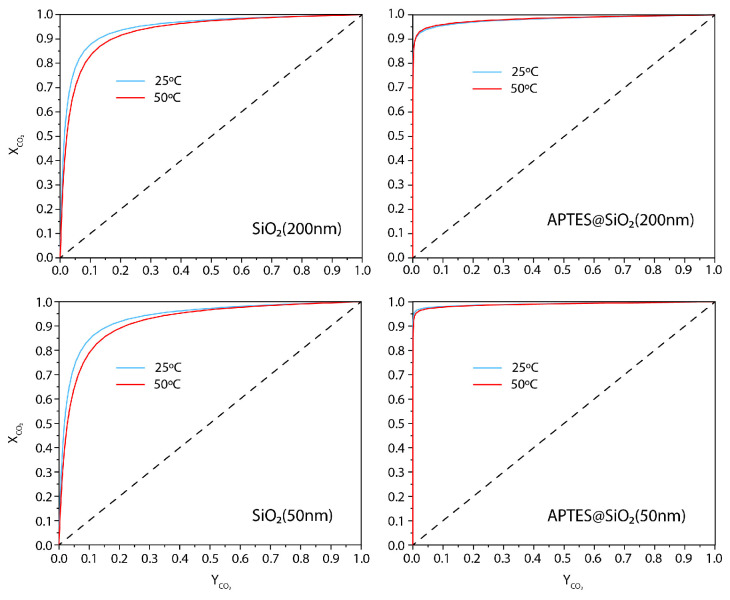
Prediction of the equilibrium composition of the adsorbed phase (*x*_CO_2__) as a function of the CO_2_ mole fraction in the gas phase (*y*_CO_2__) in CO_2_/N_2_ mixtures at 750 mmHg and at 25 and 50 °C.

The prediction of the equilibrium compositions of the adsorbed phase, *x_i_* (*i* = CO_2_ in this case) as a function of the CO_2_ molar fraction in the gas phase (*y_i_*) in CO_2_/N_2_ binary mixtures at atmospheric pressure (750 mmHg) and at 25 and 50 °C were calculated using the procedure explained in the Experimental Section and represented in Figure 14 for the four samples studied in this work. As can be seen, and as is to be expected from the different shape and adsorption capacities shown by the samples for these gases (Figure 13), CO_2_ is strongly concentrated in the adsorbed phase due to its higher adsorption potential. For all samples, the molar fraction of the adsorbed phase, *x_i_*, will be made up mainly of CO_2_, even in dilute mixtures of CO_2_ (low *y*_CO_2__). This is especially noticeable in the case of functionalized samples, and especially for sample SiO_2_ (50 nm)@APTES, where the adsorbed phase will be formed practically only by CO_2_ at any composition of the gas phase. These results suggest that functionalized samples would have an exceptional behavior in the separation of these gases, especially in diluted streams.

This conclusion is corroborated by the expected amount of adsorbed moles deter-mined with Equations (7) and (8) from the mole fractions predicted by IAST (Figure 14). The predicted adsorbed amount of both gases as function of the CO_2_ mole fraction in the phase gas at 750 mmHg and both temperatures are plotted in Figure S4. The amount of N_2_ adsorbed on non-functionalized SiO_2_ nanoparticles from diluted mixtures (i.e., CO_2_ molar fractions, *y*_CO_2__ < 0.2), although very small, is not negligible and therefore the separation of both gases will not be entirely effective. On the other hand, in the case of the functionalized samples, the amount of N_2_ adsorbed is negligible compared to the amount of CO_2_ adsorbed at any mole fraction in the gas phase. Therefore, a good separation of both gases could be carried out with these adsorbents.

Finally, the selectivity factors, S_1,2_ (where 1 = CO_2_ and 2 = N_2_), were calculated using Equation (9) and represented in Figures S5 and S6. The selectivity factors were calculated for three different compositions of the mixture of both gases (Figure S5). The theoretical maximum value of S_1,2_ is given when the gas consists of pure nitrogen (*y*_CO_2__ = 0) and the theoretical minimum one for the case that the gas is pure CO_2_ (*y*_CO_2__ = 1). Any binary mixture of these gases will possess selectivity factors that are within these limits, as shown in the case of a mixture with a 0.2 molar ratio of CO_2_ in N_2_. For all the samples, the selectivity factors, S_1,2_, decrease when increasing both the pressure and the temperature. In the case of pristine SiO_2_ nanoparticles, the larger ones (200 nm) displayed higher selectivity factors than the smaller ones. At 750 mmHg, the selectivity factors for SiO_2_ (200 nm) and SiO_2_ (50 nm) nanoparticles vary in the range 40–71 and 30–55 at 25 °C and between 35–46 and 26–35 at 50 °C, respectively. These values are comparable, or even higher, to those achieved by other types of sorbents studied in the separation of binary mixtures of these gases. For example, selectivity factors at room temperature and *y*_CO_2__ ~0.15 were reported in the range 26–78 for microporous polymers [55,56,57]. In the case of nitrogen-doped carbon materials, selectivity factors at room temperature also ranged between 15 and 70 [58,59,60,61,62,63], achieving values up to 110 for the best case reported recently [64]. Metalorganic framework (MOFs) and zeolitic imidazolate frameworks (ZIFs) were also studied in this application and values of selectivity lower than 67 have been reported [65,66,67]. Finally, selectivity factors ranging between 10 and 100 can be found in the literature in the case of zeolites and silica materials for both pristine and functionalized amines [68,69,70,71].

In the case of functionalized samples, the selectivity factors are higher for the sample prepared from the smallest nanoparticles, APTES@SiO_2_ (50 nm). This is due to the higher degree of functionalization achieved with this particular material, as it was demonstrated previously, showing higher heat adsorption and adsorption capacity, favoring a better performance in the CO_2_ separation. Both samples displayed outstanding selectivity factors, with maximum theoretical values (i.e., at *y*_CO_2__ = 0) at atmospheric pressure and 25 °C of about 6.6 × 10^3^ and 15 × 10^3^ for the samples APTES@SiO_2_ (200 nm) and APTES@SiO_2_ (50 nm), respectively. These values also remain exceptionally high at 50 °C (see Figure S5). J. A. Cecilia et al. [72] reported similar selectivity factor for CO_2_/N_2_ separation with mesoporous silica SBA-15, also grafted with APTES. As an example of how these functionalized nanoparticles would behave for the capture (i.e., separation) of CO_2_ from N_2_ in a post-combustion gas stream, Figure S6 shows the variation of the selectivity factors at a pressure of 750 mmHg and at two different temperatures, 25 (dashed line) and 50 °C (solid line), in a range of mole fractions that covers values typical of post-combustion processes (e.g., *y*_CO_2__ < 0.15–0.20) [73]. The selectivity factor decreases with increasing the molar fraction of CO_2_ in the mixture and the temperature of the gas, as just mentioned. As can be seen in the figure, for CO_2_ molar fractions < 0.2, S_1,2_ remain above 260 for the 50 nm size functionalized nanoparticles and at values greater than 130 for the larger functionalized nanoparticles. These values are much higher than those reported by other adsorbents as was explained above and, to the best of our knowledge, only two materials with similar selectivity factors have been reported. Thus, K. Hwang et al. reported a CO_2_/N_2_ (molar ratio of 0.15) selectivity of 186 for the molecular sieve zeolite 4A [74] and J. Park et al. reported a selectivity factor of 196 for separation of a mixture 0.15 molar of CO_2_/N_2_ at 1 atm and 25 °C for a MOF functionalized with ethylenediaminetetraacetic acid (EDTA), further reacted with ethylenediamine (ED), and finally, reduced with lithium aluminum hydride [75].

## 4. Concluding Discussion

Amine functionalized silica nanoparticles of 50 nm size were successfully synthesized by means of APTES ligands. Owing to our previous results in 200 nm size SiO_2_NPs, due to APTES being the most efficient ligand for CO_2_ sequestration, we here compared both species (50 vs. 200 nm), resulting in a marked enhancement of the CO_2_ sequestration by a factor of 1.4. The organic loading of the SiO_2_ (50 nm) nanoparticles was calculated by accurate colorimetric analysis, leading to 0.78 mmol (APTES)/g SiO_2_ (50 nm), which represents a 4-fold increase compared to the coating of the 200 nm sized. The organic loadings were also confirmed by TGA analysis.

Temperature-swing adsorption-desorption cycles demonstrated a stable working capacity of the materials without any evident loss in the CO_2_ sequestration capacity and amine efficiency. Additionally, IAST demonstrated excellent CO_2_/N_2_ selectivity for both pristine samples (SiO_2_ (50 nm) and SiO_2_ (200 nm)) and their APTES functionalized counterparts.

In summary, we demonstrated that SiO_2_ (50 nm) nanoparticles benefit from their tuneability, selectivity, low cost, fair CO_2_ uptake (1.14 mmol/g), and accessible chemical functionalization. All these properties make them interesting candidates as sorbent “active sites”, which can be incorporated in an ordered-fashion molecular framework such as multi-walled carbon nanotubes (MWCNTs) [53].

## Figures and Tables

**Table 1:** Porous textural parameters derived from the N_2_ adsorption isotherms at −196 °C and the CO_2_ adsorption isotherms at 0 °C of the four SiO_2_NPs of study. A few classical sorbent examples reported in the literature were also included.

Material	N_2_ Adsorption				CO_2_ Adsorption	
	S_BET_	S_ext_	V_T_	V_DR_, N_2_	V_DR_, CO_2_	Total CO_2_ Adsorption
	(m^2^/g)	(m^2^/g)	(cm^3^/g)	(cm^3^/g)	(cm^3^/g)	(mmol/g) ^a^
SiO_2_ (50 nm)	129	76	0.25	0.06	0.11	0.94 ^b^
APTES@SiO_2_ (50 nm)	41	36	0.13	0.02	0.09	1.14 ^b^
SiO_2_ (200 nm)	20	20	0.03	0.006	0.13	1.00 ^b^
APTES@SiO_2_ (200 nm)	13	13	0.02	0.005	0.10	1.05 ^b^
MOFs [50]	1387	-	-	-	-	4.88 ^b^
MOFs [51]	516	-	0.26	-	-	2.75 ^b^
N-doped porous C [52]	860.4	-	-	-	-	4.65 ^c^
N-doped porous C [53]	1017	-	-	-	-	2.63 ^c^
N-doped porous C [54]	1353	-	-	-	-	5.67 ^c^

^a^ Adsorption capacity at temperature of 25 °C; ^b^ CO_2_ adsorption capacity at 1 bar; ^c^ CO_2_ adsorption capacity at 5 bar.

## Data Availability

Data is contained within the article.

## Supplementary Materials

The following are available online at https://www.mdpi.com/article/10.3390/nano11112893/s1, Figure S1: Some of the different simulations carried out using the Equation (S1) to achieve the optimum fitting employing the minimum number of *a_i_* and *b_j_* parameters. The isotherms were measured at 0 and 25 °C using (A) 5*a_i_* and 2*b_j_* parameters; (B) 7*a_i_* and 3*b_j_* parameters; (C) 10*a_i_* and 5*b_j_* parameters; (D) 8*a_i_* and 3*b_j_* parameters. Figure S2: CO_2_ adsorption/desorption isotherms at 0, 25 and 50 °C for pristine and functionalized SiO_2_ nanoparticles of 50 and 200 nm. Figure S3: IR spectra of 50 nm size (a,b) SiO_2_ (50 nm), before (navy blue) and after (green) CO_2_/N_2_ cycles, and (c,d) APTES@SiO_2_ (50 nm), before (red) and after (pink) CO_2_/N_2_ cycles. Table S1: Henry’s constant corresponding to the lineal fit of the N_2_ adsorption isotherms at 25 and 50 °C on pristine and functionalized SiO_2_ nanoparticles of 50 and 200 nm size. Figure S4: Prediction of the amount of CO_2_ and N_2_ adsorbed as a function of the CO_2_ mole fraction in the gas phase (*y*_CO_2__) in CO_2_/N_2_ mixtures at 750 mmHg and at 25 and 50 °C. Figure S5: Variation of the selectivity factors, S_1,2_, of CO_2_ over N_2_ in a mixture of both gases as function of the gas composition at 25 and 50 *°C* and different pressures. S*_min_* = minimum value of S_1,2_ at *y*_CO_2__ = 1 and S*_max_* = maximum value of S_1,2_ at *y*_CO_2__ = 0. Figure S6: Variation of the selectivity factors, *S*_1,2_, of CO_2_ over N_2_ as a function of the mole fraction of CO_2_ (*y*_CO_2__) at 750 mmHg and at 25 (dashed line) and 50 °C (solid line) on the functionalized nanoparticles.
