# Supplementary material for: APTES-Based Silica Nanoparticles as a Potential Modifier for the Selective Sequestration of CO2 Gas Molecules
Source: Nanomaterials (Basel). 2021 Oct 29;11(11):2893. doi: 10.3390/nano11112893 (PMC8620991; doi:10.3390/nano11112893)
Supplement: Supplementary file 1 [file nanomaterials-11-02893-s001.zip › nanomaterials-1382622-supplementary.pdf]

Supporting information

for

APTES based silica nanoparticles as a potential modifier for the selective sequestration of CO_2 gas molecules

Eduardo J. Cueto-Díaz[1,*], Alberto Castro Muñiz[2], Fabián Suárez-García[2], Santos Gálvez-Martínez[1], Mª Carmen Torquemada-Vico[3], Mª Pilar Valles González[4] and Eva Mateo-Martí [1].

[1]Centro de Astrobiología, (INTA-CSIC), Ctra. Ajalvir, Km. 4, 28850, Torrejón de Ardoz, Madrid, Spain; sgalvez@cab.inta-csic.es (S.G.-M.); mateome@cab.inta-csic.es (E.M.-M.)
[2]Instituto de Ciencia y Tecnología del Carbono (INCAR-CSIC), C/ Francisco Pintado Fe, 26, 33011 Oviedo, Spain; alberto@incar.csic.es (A.C.M.); fabian@incar.csic.es (F.S.-G.)
[3]Departamento de Óptica Espacial, Instituto Nacional de Técnica Aeroespacial, Ctra. Ajalvir, Km. 4, 28850, Torrejón de Ardoz, Madrid, Spain; torquemadavm@inta.es
[4]Departamento de Materiales y Estructuras, Instituto Nacional de Técnica Aeroespacial, Ctra. Ajalvir, Km. 4, 28850, Torrejón de Ardoz, Madrid, Spain; vallesgp@inta.es
* Correspondence: ecueto@cab.inta-csic.es

Table of Contents

A. Heat of adsorption	p. 1
B. CO_2 adsorption/desorption isotherms	p. 2
C. FT-IR spectra	p. 3
D. Selectivity	p. 3
E. References	p. 6

1. Heat of adsorption

Isosteric heat (enthalpy in this case) of adsorption was calculated using the Virial equation. This method involved the simultaneous fit of two adsorption isotherms with the same fitting parameters a_i and b_j (see equation 1). The number of fitting parameters must also be optimized during the fit. So, several simulations have to be carried out in order to achieve the optimum fitting trying to use the minimum number of a_i and b_j parameters. In practice, five a_i and two b_j parameters are a good option to start for the fit and more parameters should be added until the final fit does not improve more [1,2].

$$\ln P = \ln n + \frac{1}{T}\sum_{i=0}^{m} a_i n^i + \sum_{j=0}^{n} b_j n^j \qquad \text{(S1)}$$

Where, P is the pressure, n in the adsorbed amount, T is the temperature and a_i and b_j are the fitting parameters.

The isosteric heat of adsorption (Q_{st}) can be calculated from the a_i parameters (Equation 2):

$$Q_{st} = -R\sum_{i=0}^{m} a_i n^i \qquad \text{(S2)}$$

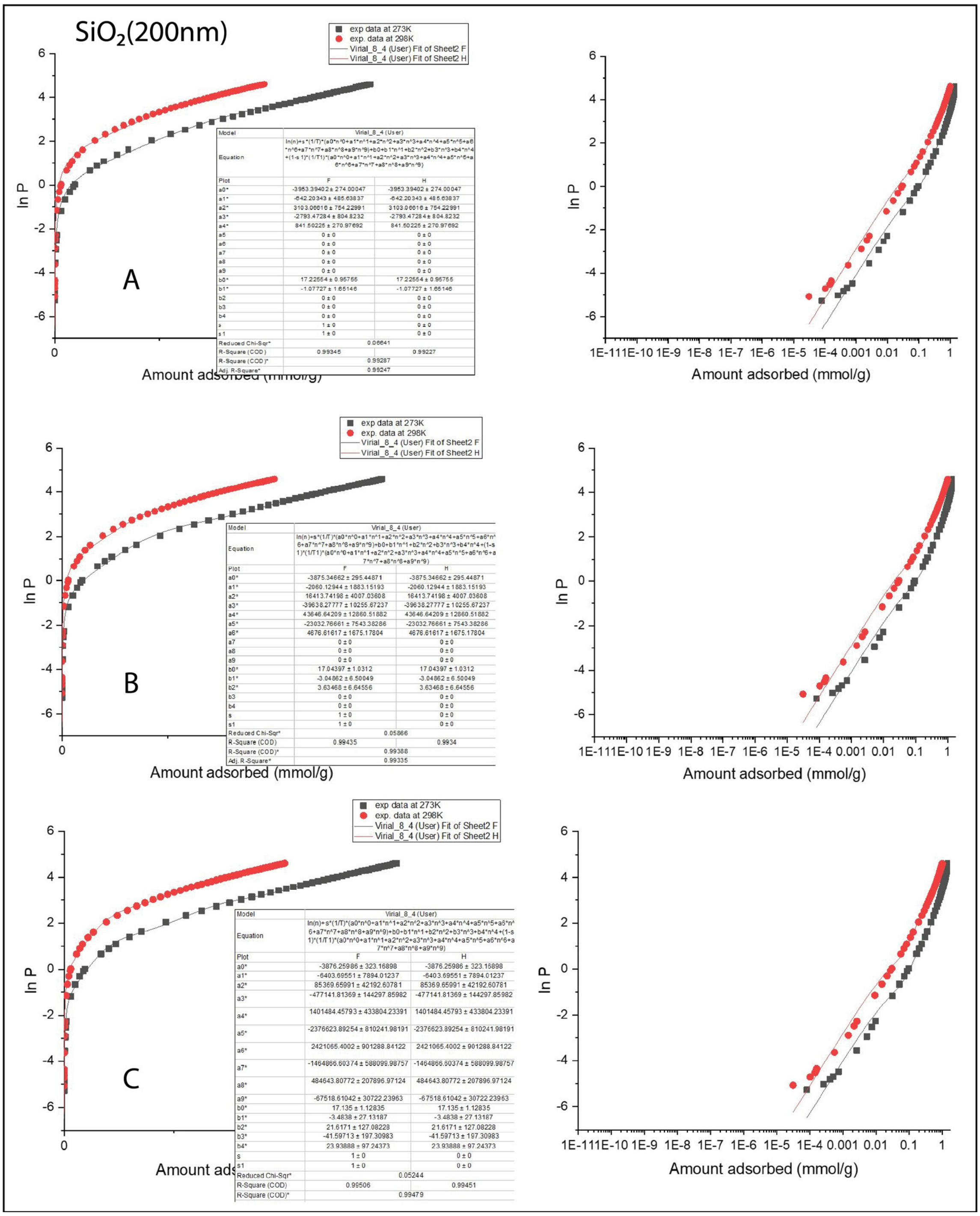

A

Model	Virial_8_4 (User)	
Equation	ln(n)+s*(1/T)*(a0*n^0+a1*n^1+a2*n^2+a3*n^3+a4*n^4+a5*n^5+a6*n^6+a7*n^7+a8*n^8+a9*n^9)+b0+b1*n^1+b2*n^2+b3*n^3+b4*n^4+(1-s1)*(1/T1)*(a0*n^0+a1*n^1+a2*n^2+a3*n^3+a4*n^4+a5*n^5+a6*n^6+a7*n^7+a8*n^8+a9*n^9)	
Plot	F	H
a0*	-3953.39402 ± 274.00047	-3953.39402 ± 274.00047
a1*	-642.20343 ± 485.63837	-642.20343 ± 485.63837
a2*	3103.06616 ± 754.22991	3103.06616 ± 754.22991
a3*	-2793.47284 ± 804.8232	-2793.47284 ± 804.8232
a4*	841.50225 ± 270.97692	841.50225 ± 270.97692
a5	0 ± 0	0 ± 0
a6	0 ± 0	0 ± 0
a7	0 ± 0	0 ± 0
a8	0 ± 0	0 ± 0
a9	0 ± 0	0 ± 0
b0*	17.22554 ± 0.95755	17.22554 ± 0.95755
b1*	-1.07727 ± 1.65146	-1.07727 ± 1.65146
b2	0 ± 0	0 ± 0
b3	0 ± 0	0 ± 0
b4	0 ± 0	0 ± 0
s	1 ± 0	0 ± 0
s1	1 ± 0	0 ± 0
Reduced Chi-Sqr*	0.06641	
R-Square (COD)	0.99345	0.99227
R-Square (COD)*	0.99287	
Adj. R-Square*	0.99247	

B

Model	Virial_8_4 (User)	
Equation	ln(n)+s*(1/T)*(a0*n^0+a1*n^1+a2*n^2+a3*n^3+a4*n^4+a5*n^5+a6*n^6+a7*n^7+a8*n^8+a9*n^9)+b0+b1*n^1+b2*n^2+b3*n^3+b4*n^4+(1-s1)*(1/T1)*(a0*n^0+a1*n^1+a2*n^2+a3*n^3+a4*n^4+a5*n^5+a6*n^6+a7*n^7+a8*n^8+a9*n^9)	
Plot	F	H
a0*	-3875.34662 ± 295.44871	-3875.34662 ± 295.44871
a1*	-2060.12944 ± 1883.15193	-2060.12944 ± 1883.15193
a2*	16413.74198 ± 4007.03608	16413.74198 ± 4007.03608
a3*	-39638.27777 ± 10255.67237	-39638.27777 ± 10255.67237
a4*	43646.64209 ± 12860.51882	43646.64209 ± 12860.51882
a5*	-23032.76661 ± 7543.38286	-23032.76661 ± 7543.38286
a6*	4676.61617 ± 1675.17804	4676.61617 ± 1675.17804
a7	0 ± 0	0 ± 0
a8	0 ± 0	0 ± 0
a9	0 ± 0	0 ± 0
b0*	17.04397 ± 1.0312	17.04397 ± 1.0312
b1*	-3.04862 ± 6.50049	-3.04862 ± 6.50049
b2*	3.63468 ± 6.64556	3.63468 ± 6.64556
b3	0 ± 0	0 ± 0
b4	0 ± 0	0 ± 0
s	1 ± 0	0 ± 0
s1	1 ± 0	0 ± 0
Reduced Chi-Sqr*	0.05866	
R-Square (COD)	0.99435	0.9934
R-Square (COD)*	0.99388	
Adj. R-Square*	0.99335	

C

Model	Virial_8_4 (User)	
Equation	ln(n)+s*(1/T)*(a0*n^0+a1*n^1+a2*n^2+a3*n^3+a4*n^4+a5*n^5+a6*n^6+a7*n^7+a8*n^8+a9*n^9)+b0+b1*n^1+b2*n^2+b3*n^3+b4*n^4+(1-s1)*(1/T1)*(a0*n^0+a1*n^1+a2*n^2+a3*n^3+a4*n^4+a5*n^5+a6*n^6+a7*n^7+a8*n^8+a9*n^9)	
Plot	F	H
a0*	-3876.25986 ± 323.16898	-3876.25986 ± 323.16898
a1*	-6403.69551 ± 7894.01237	-6403.69551 ± 7894.01237
a2*	85369.65991 ± 42192.60781	85369.65991 ± 42192.60781
a3*	-477141.81369 ± 144297.85982	-477141.81369 ± 144297.85982
a4*	1401484.45793 ± 433804.23391	1401484.45793 ± 433804.23391
a5*	-2376623.89254 ± 810241.98191	-2376623.89254 ± 810241.98191
a6*	2421065.4002 ± 901288.84122	2421065.4002 ± 901288.84122
a7*	-1464866.60374 ± 588099.98757	-1464866.60374 ± 588099.98757
a8*	484643.80772 ± 207896.97124	484643.80772 ± 207896.97124
a9*	-67518.61042 ± 30722.23963	-67518.61042 ± 30722.23963
b0*	17.135 ± 1.12835	17.135 ± 1.12835
b1*	-3.4838 ± 27.13187	-3.4838 ± 27.13187
b2*	21.6171 ± 127.08228	21.6171 ± 127.08228
b3*	-41.59713 ± 197.30983	-41.59713 ± 197.30983
b4*	23.93888 ± 97.24373	23.93888 ± 97.24373
s	1 ± 0	0 ± 0
s1	1 ± 0	0 ± 0
Reduced Chi-Sqr*	0.05244	
R-Square (COD)	0.99506	0.99451
R-Square (COD)*	0.99479	

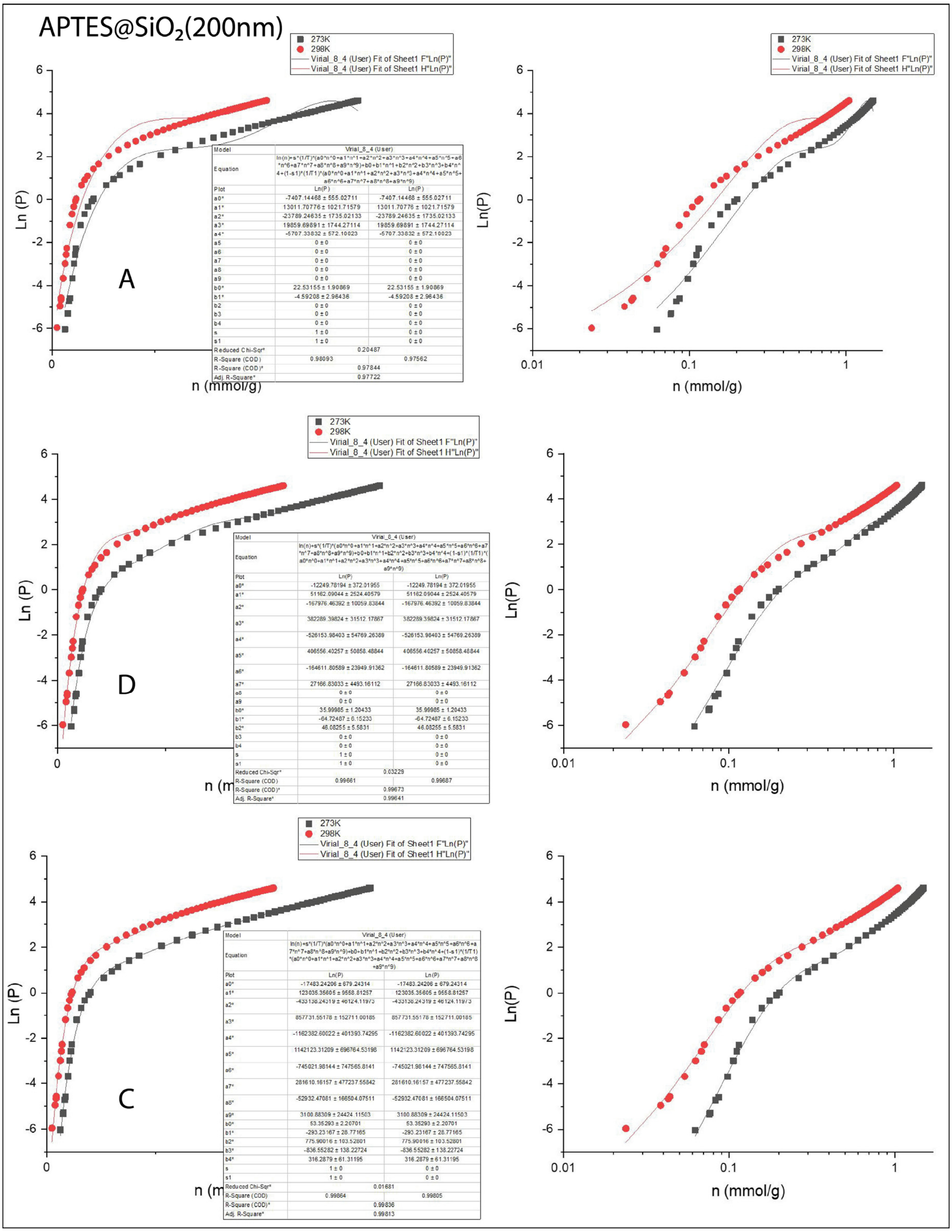

Model	Virial_8_4 (User)	
Equation	ln(n)+s*(1/T)*(a0*n^0+a1*n^1+a2*n^2+a3*n^3+a4*n^4+a5*n^5+a6*n^6+a7*n^7+a8*n^8+a9*n^9)+b0+b1*n^1+b2*n^2+b3*n^3+b4*n^4+(1-s1)*(1/T1)*(a0*n^0+a1*n^1+a2*n^2+a3*n^3+a4*n^4+a5*n^5+a6*n^6+a7*n^7+a8*n^8+a9*n^9)	
Plot	Ln(P)	Ln(P)
a0*	-7407.14468 ± 555.02711	-7407.14468 ± 555.02711
a1*	13011.70776 ± 1021.71579	13011.70776 ± 1021.71579
a2*	-23789.24635 ± 1735.02133	-23789.24635 ± 1735.02133
a3*	19859.69891 ± 1744.27114	19859.69891 ± 1744.27114
a4*	-5707.33832 ± 572.10023	-5707.33832 ± 572.10023
a5	0 ± 0	0 ± 0
a6	0 ± 0	0 ± 0
a7	0 ± 0	0 ± 0
a8	0 ± 0	0 ± 0
a9	0 ± 0	0 ± 0
b0*	22.53155 ± 1.90869	22.53155 ± 1.90869
b1*	-4.59208 ± 2.96436	-4.59208 ± 2.96436
b2	0 ± 0	0 ± 0
b3	0 ± 0	0 ± 0
b4	0 ± 0	0 ± 0
s	1 ± 0	0 ± 0
s1	1 ± 0	0 ± 0
Reduced Chi-Sqr*	0.20487	
R-Square (COD)	0.98093	0.97562
R-Square (COD)*	0.97844	
Adj. R-Square*	0.97722	

Model	Virial_8_4 (User)	
Equation	ln(n)+s*(1/T)*(a0*n^0+a1*n^1+a2*n^2+a3*n^3+a4*n^4+a5*n^5+a6*n^6+a7*n^7+a8*n^8+a9*n^9)+b0+b1*n^1+b2*n^2+b3*n^3+b4*n^4+(1-s1)*(1/T1)*(a0*n^0+a1*n^1+a2*n^2+a3*n^3+a4*n^4+a5*n^5+a6*n^6+a7*n^7+a8*n^8+a9*n^9)	
Plot	Ln(P)	Ln(P)
a0*	-12249.78194 ± 372.01955	-12249.78194 ± 372.01955
a1*	51162.09044 ± 2524.40579	51162.09044 ± 2524.40579
a2*	-167976.46392 ± 10059.83844	-167976.46392 ± 10059.83844
a3*	382289.39824 ± 31512.17867	382289.39824 ± 31512.17867
a4*	-526153.98403 ± 54769.26389	-526153.98403 ± 54769.26389
a5*	406556.40257 ± 50858.48844	406556.40257 ± 50858.48844
a6*	-164611.80589 ± 23949.91362	-164611.80589 ± 23949.91362
a7*	27166.83033 ± 4493.16112	27166.83033 ± 4493.16112
a8	0 ± 0	0 ± 0
a9	0 ± 0	0 ± 0
b0*	35.99985 ± 1.20433	35.99985 ± 1.20433
b1*	-64.72487 ± 6.15233	-64.72487 ± 6.15233
b2*	46.08255 ± 5.5831	46.08255 ± 5.5831
b3	0 ± 0	0 ± 0
b4	0 ± 0	0 ± 0
s	1 ± 0	0 ± 0
s1	1 ± 0	0 ± 0
Reduced Chi-Sqr*	0.03229	
R-Square (COD)	0.99661	0.99687
R-Square (COD)*	0.99673	
Adj. R-Square*	0.99641	

Model	Virial_8_4 (User)	
Equation	ln(n)+s*(1/T)*(a0*n^0+a1*n^1+a2*n^2+a3*n^3+a4*n^4+a5*n^5+a6*n^6+a7*n^7+a8*n^8+a9*n^9)+b0+b1*n^1+b2*n^2+b3*n^3+b4*n^4+(1-s1)*(1/T1)*(a0*n^0+a1*n^1+a2*n^2+a3*n^3+a4*n^4+a5*n^5+a6*n^6+a7*n^7+a8*n^8+a9*n^9)	
Plot	Ln(P)	Ln(P)
a0*	-17483.24206 ± 679.24314	-17483.24206 ± 679.24314
a1*	123035.35605 ± 9558.81257	123035.35605 ± 9558.81257
a2*	-433138.24319 ± 46124.11973	-433138.24319 ± 46124.11973
a3*	857731.55178 ± 152711.00185	857731.55178 ± 152711.00185
a4*	-1162382.60022 ± 401393.74295	-1162382.60022 ± 401393.74295
a5*	1142123.31209 ± 696764.53198	1142123.31209 ± 696764.53198
a6*	-745021.98144 ± 747565.8141	-745021.98144 ± 747565.8141
a7*	281610.16157 ± 477237.55842	281610.16157 ± 477237.55842
a8*	-52932.47081 ± 166504.07511	-52932.47081 ± 166504.07511
a9*	3100.88309 ± 24424.11503	3100.88309 ± 24424.11503
b0*	53.35293 ± 2.20701	53.35293 ± 2.20701
b1*	-293.23167 ± 28.77165	-293.23167 ± 28.77165
b2*	775.90016 ± 103.52801	775.90016 ± 103.52801
b3*	-836.55282 ± 138.22724	-836.55282 ± 138.22724
b4*	316.2879 ± 61.31195	316.2879 ± 61.31195
s	1 ± 0	0 ± 0
s1	1 ± 0	0 ± 0
Reduced Chi-Sqr*	0.01681	
R-Square (COD)	0.99864	0.99805
R-Square (COD)*	0.99836	
Adj. R-Square*	0.99813	

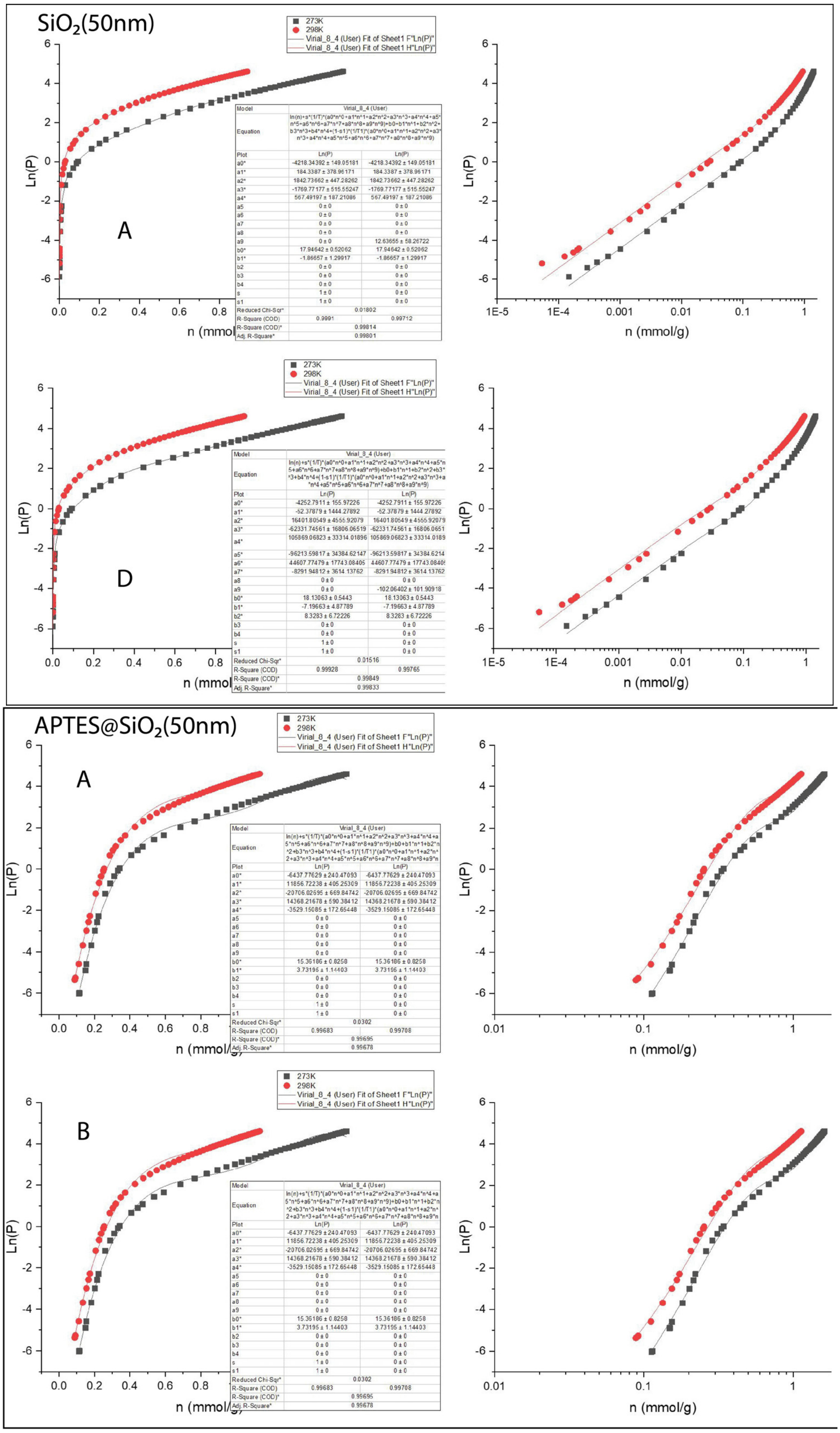

Figure S1. Some of the different simulations carried out using the Equation S1 to achieve the optimum fitting employing the minimum number of a_i and b_j parameters. The isotherms were measured at 0 and 25°C using (A) 5a_i and 2b_j parameters; (B) 7a_i and 3b_j parameters; (C) 10a_i and 5b_j parameters and (D) 8a_i and 3b_j parameters.

2. CO_2 adsorption/desorption isotherms

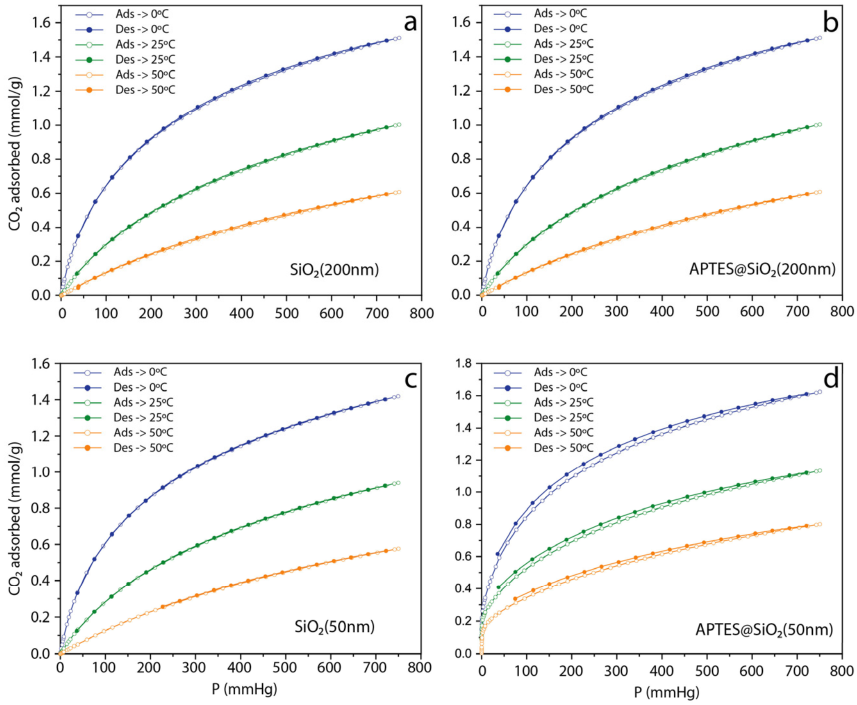

Figure S2. CO_2 adsorption/desorption isotherms at 0, 25 and 50°C for pristine and functionalized SiO_2 nanoparticles of 50 and 200 nm.

3. FT-IR spectra

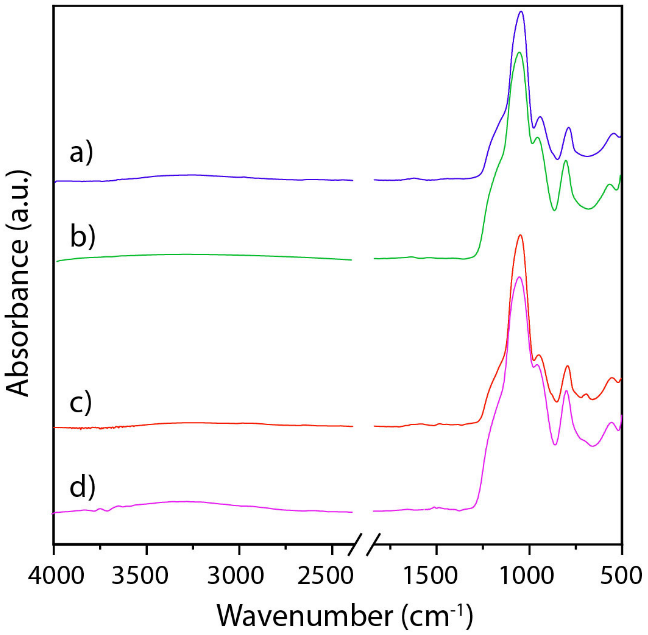

Figure S3. IR spectra of 50 nm size (a,b) SiO_2(50nm), before (navy blue) and after (green) CO_2/N_2 cycles, and (c,d) SiO_2(50nm)@APTES, before (red) and after (pink) CO_2/N_2 cycles.

4. Selectivity

Table S1. Henry's constants corresponding to the lineal fit of the N_2 adsorption isotherms at 25 and 50°C on pristine and functionalized SiO_2 nanoparticles of 50 and 200 nm of size.

Sample	N_2 at 25°C	N_2 at 50°C

	K_H (mmol/g/mmHg)	R^2	K_H (mmol/g/mmHg)	R^2
SiO_2(200nm)	$5.43 \cdot 10^{-5}$	0.9999	$3.07 \cdot 10^{-5}$	0.9949
SiO_2(200nm)@APTES	$5.63 \cdot 10^{-5}$	0.9970	$2.93 \cdot 10^{-5}$	0.9905
SiO_2(50nm)	$6.82 \cdot 10^{-5}$	0.9999	$3.94 \cdot 10^{-5}$	0.9999
SiO_2(50nm)@APTES	$4.94 \cdot 10^{-5}$	0.9927	$3.25 \cdot 10^{-5}$	0.9744

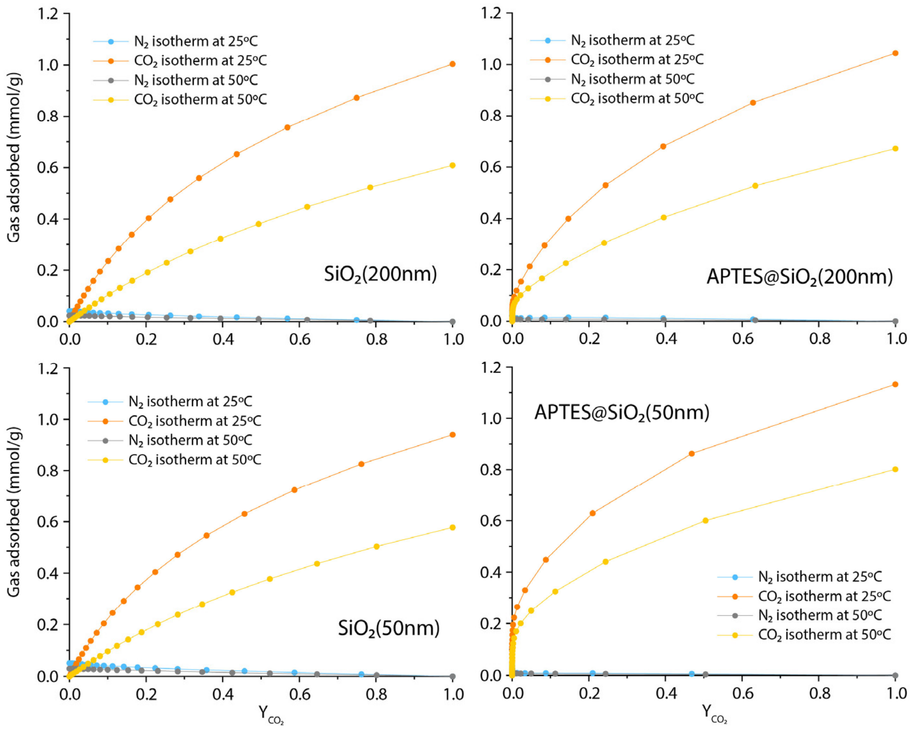

Figure S4. Prediction of the amount of CO_2 and N_2 adsorbed as a function of the CO_2 mole fraction in the gas phase (y_{CO2}) in CO_2/N_2 mixtures at 750 mmHg and at 25 and 50°C.

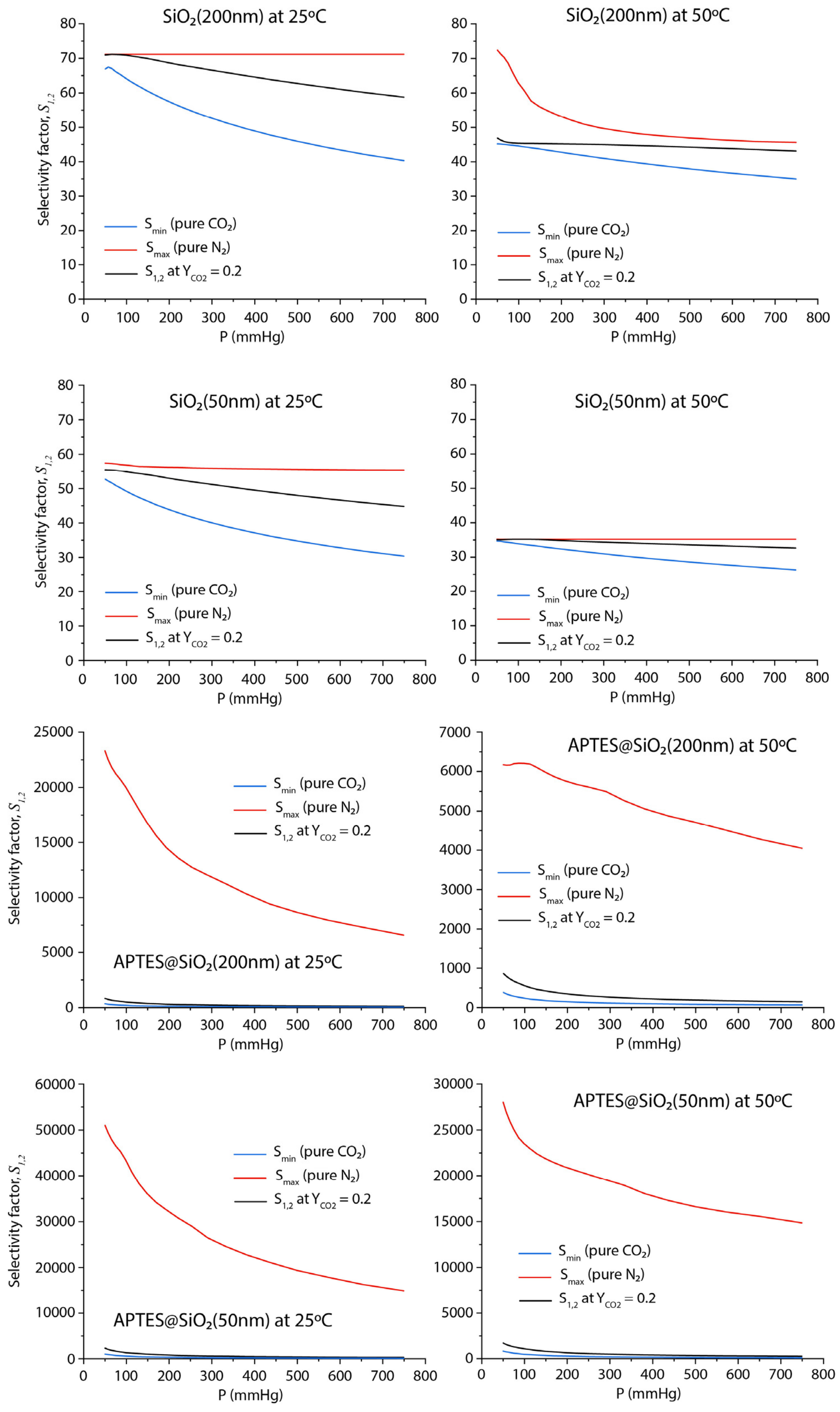

Figure S5. Variation of the selectivity factors, $S_{1,2}$, of CO_2 over N_2 in a mixture of both gases as function of the gas composition at 25 and 50°C and different pressures. S_{min} = minimum value of $S_{1,2}$ at y_{CO2} = 1 and S_{max} = maximum value of $S_{1,2}$ at y_{CO2} = 0. .

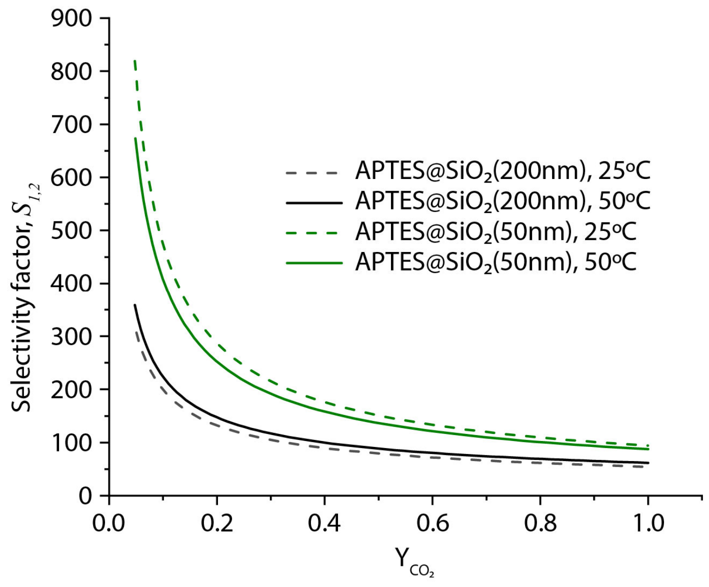

Figure S6. Variation of the selectivity factors $S_{1,2}$, of CO_2 over N_2 as function of the mole fraction of CO_2 (y_{CO2}) at 750 mmHg and at 25 (dashed line) and 50°C (solid line) on the functionalized nanoparticles.

References

1. Czepirski, L.; JagieŁŁo, J. Virial-type thermal equation of gas—solid adsorption. *Chemical Engineering Science* **1989**, *44*, 797-801, doi:https://doi.org/10.1016/0009-2509(89)85253-4.
2. Nuhnen, A.; Janiak, C. A practical guide to calculate the isosteric heat/enthalpy of adsorption via adsorption isotherms in metal–organic frameworks, MOFs. *Dalton Transactions* **2020**, *49*, 10295-10307, doi:10.1039/D0DT01784A.
